# Epigenetic Research in Stem Cell Bioengineering—Anti-Cancer Therapy, Regenerative and Reconstructive Medicine in Human Clinical Trials

**DOI:** 10.3390/cancers12041016

**Published:** 2020-04-21

**Authors:** Claudia Dompe, Krzysztof Janowicz, Greg Hutchings, Lisa Moncrieff, Maurycy Jankowski, Mariusz J. Nawrocki, Małgorzata Józkowiak, Paul Mozdziak, Jim Petitte, Jamil A. Shibli, Marta Dyszkiewicz-Konwińska, Małgorzata Bruska, Hanna Piotrowska-Kempisty, Bartosz Kempisty, Michał Nowicki

**Affiliations:** 1 Department of Histology and Embryology, Poznan University of Medical Sciences, 60-781 Poznan, Poland; u16cd16@abdn.ac.uk (C.D.); l.moncrieff.16@abdn.ac.uk (L.M.); mnowicki@ump.edu.pl (M.N.); 2 The School of Medicine, Medical Sciences and Nutrition, University of Aberdeen, Aberdeen AB25 2ZD, UK; krzysztof.janowicz.16@abdn.ac.uk (K.J.); g.hutchings.16@abdn.ac.uk (G.H.); 3 Department of Anatomy, Poznan University of Medical Sciences, 60-781 Poznan, Poland; mjankowski@ump.edu.pl (M.J.); mjnawrocki@ump.edu.pl (M.J.N.); m.dyszkiewicz@ump.edu.pl (M.D.-K.); mbruska@ump.edu.pl (M.B.); 4 Department of Toxicology, Poznan University of Medical Sciences, 61-631 Poznan, Poland; malgorzata.jozkowiak@gmail.com (M.J.); hpiotrow@ump.edu.pl (H.P.-K.); 5 Physiology Graduate Program, North Carolina State University, Raleigh, NC 27695, USA; pemozdzi@ncsu.edu; 6 Prestage Department of Poultry Science, North Carolina State University, Raleigh, NC 27695, USA; jnppo@ncsu.edu; 7 Department of Periodontology and Oral Implantology, Dental Research Division, University of Guarulhos, São Paulo 07023-070, Brazil; jashibli@yahoo.com; 8 Department of Biomaterials and Experimental Dentistry, Poznan University of Medical Sciences, 61 701 Poznan, Poland; 9 Department of Obstetrics and Gynaecology, University Hospital and Masaryk University, 602 00 Brno, Czech Republic; 10 Department of Veterinary Surgery, Institute of Veterinary Medicine, Nicolaus Copernicus University in Torun, 87 100 Torun, Poland

**Keywords:** epigenetics, cancer, stem cells, regenerative medicine, reconstructive medicine

## Abstract

The epigenome denotes all the information related to gene expression that is not contained in the DNA sequence but rather results from chemical changes to histones and DNA. Epigenetic modifications act in a cooperative way towards the regulation of gene expression, working at the transcriptional or post-transcriptional level, and play a key role in the determination of phenotypic variations in cells containing the same genotype. Epigenetic modifications are important considerations in relation to anti-cancer therapy and regenerative/reconstructive medicine. Moreover, a range of clinical trials have been performed, exploiting the potential of epigenetics in stem cell engineering towards application in disease treatments and diagnostics. Epigenetic studies will most likely be the basis of future cancer therapies, as epigenetic modifications play major roles in tumour formation, malignancy and metastasis. In fact, a large number of currently designed or tested clinical approaches, based on compounds regulating epigenetic pathways in various types of tumours, employ these mechanisms in stem cell bioengineering.

## 1. Introduction

In recent years, significant progress has been made towards understanding the mechanisms that modify the genome in response to environmental stimuli. Historically, the focus has been directed towards genetic mutations in DNA. However, the importance of epigenetic modification has recently been outlined as a possible cause for environmentally induced diseases [1]. Epigenetics studies the mitotically and meiotically heritable modifications of gene expression that do not involve the primary sequence of the DNA. This field is based on parent cell–progeny transmission of alterations regulating gene transcription, leading to changes in cell lineage, function and fate [2]. The main mechanisms of epigenetic regulation include DNA methylation, histone modifications, promoter–enhancer interactions, as well as non-coding RNA-mediated regulation [3].

It has been proven that these distinct types of genetic mechanisms are deeply involved in a range of major disease groups, which can be inherited or somatically acquired and the treatment of which remains a challenge in the modern medicine. Notable examples include cardiovascular diseases, neurodegenerative disease, metabolic disorder, obesity and bone and skeletal disease [4,5,6,7,8,9]. Many disruptive epigenetic mechanisms could cause pathological occurrences and may result in modification of the chromosome, leading to learning disabilities. For example, fragile X syndrome derives from the silencing of the fragile X mental retardation 1 (*FMR1*) gene by a de novo methylation of a CGG region in its untranslated region, and immunodeficiency, centromeric region instability and facial anomalies (ICF1) syndrome is caused by alterations of the DNA methyl transferase 3b (*DNMT3b*) gene, an enzyme indispensable for methylation patterns [10,11]. Moreover, cancer stem cell development is highly connected with epigenetic aberration of progenitor genes [12]. Global alterations lead to chromosomal instability, an increased frequency of tumours and gene-specific oncogene activation, and the silencing of tumour-suppressor genes [13,14,15].

The study of epigenetic regulation is growing rapidly for the diagnosis of a disease and as targets for disease prevention strategies through the modification of epigenomes or identification of epigenetic markers. Biomedical engineers have exploited its application in search of a solution for biomedical problems. The aim of this review is to summarize the current knowledge of epigenetics, describing the most notable examples of its mechanisms, as well as its implication in disease understanding and treatment design. Additionally, a range of promising clinical studies aimed at using and regulating the epigenetic pathways will also be outlined to emphasize the importance of this field for the development of modern medicine. Finally, future research perspectives will be discussed, together with associated challenges that need to be overcome to enable the design of more complex approaches of disease treatments.

## 2. Overview of Epigenetic Modification of Genome

The three main molecular mechanisms involved in epigenetics are DNA methylation, histone modifications and non-coding RNA (ncRNA), and they are not related to a specific time of an organism’s life, but they rather continue throughout life [3]. 

### 2.1. DNA Methylation

DNA methylation is a process in which a methyl group, donated by S-adenosylmethionine, is added to the carbon-5 position of a cytosine with temporal and spatial precision [16]. This mechanism is important in CpG islands, where, mediated by DNA methyltransferases (DNMTs) and DNA demethylases, clusters of G+C can be added or erased, [17]. These CpG islands are enriched at gene promoters, which can be silenced upon methylation [18]. Three different DNMTs (DNMT1, DNMT3A, and DNMT3B) catalyse DNA methylation, and they have different functions that do not exclude one another [19]. DNA methylation can behave both as a transcription enhancer and as an inhibitor: it may repress transcription in promoter regions decreasing the expression of downstream genes, whereas in gene bodies it is correlated with active transcription [20,21]. The activity of DNA methylation can be analysed through different experimental methods depending on the results needed, including global DNA methylation, gene- or locus-specific methylation analysis and comprehensive genome-wide methylation analysis [22,23,24].

### 2.2. Histone Modification

Gene expression can also be regulated by covalent histone modification through mechanisms such as acetylation, methylation, phosphorylation, ubiquitination or sumoylation [25,26]. The crosstalk between these events and DNA methylation plays a key role in the epigenetic regulation of genome expression [27]. Histone modifications happen in a specific order and they cooperate to create a code, defined as a ‘histone code’ by Stahl and Allis and a ‘epigenetic code’ by Turner, regulating transcription and chromatin condensation, controlling accessibility to DNA [28,29]. Histone modifications may activate or inactivate neighbouring genes, alter chromatin structure and conformation or recruit transcriptional activators/suppressors by providing signals [30,31,32,33,34]. Another factor influencing the histone modification rate is the chromatin state, which can be tightly packed and non-transcribed, defined heterochromatin and or loosely packed, transcribed, and present in highly transcriptionally active cells, defined euchromatin [35]. Heterochromatin limits the access to DNA and influences the location of nucleosomes, playing a key role in transcription regulation. Moreover, the steps in histone modification are catalysed by specific enzymes. Histone acetyltransferase (HAT), histone methyltransferase (HMT), and protein arginine methyltransferase (PRMT) are responsible for ‘writing’, histone deacetylase (HDAC) and lysine demethylase (KDM) are the ‘erasers’ and, finally, the domains recognizing the modified histones are called the ‘readers’ [36]. Together with data on epigenetic regulation, histone modifications are presented in the Encyclopaedia of DNA Elements (ENCODE), created to define all the functional elements of the human genome [37].

### 2.3. Non-Coding RNA

ncRNA, classified as the small portion of genes that is transcribed but not translated into protein, also plays an important role in epigenetic regulation [38]. ncRNAs may be categorized depending on their size: small ncRNAs, including small interfering RNAs (siRNAs), microRNAs (miRNAs), Piwi-interacting RNAs (piRNAs), and long ncRNAs (lncRNAs) [39]. lncRNAs can be distinguished due to their genomic loci and/or associated DNA strands/regions as sense, anti-sense, intronic, intergenic, enhancer, or circular RNAs [40]. Depending on their function, lncRNAs can be classified in signals and decoys, which are associated with gene activation and suppression, and guides, which regulate gene expression by recruiting enzymes that modify chromatin, and scaffold, which recruits proteins which form the ribonucleoprotein complexes [41]. However, these functions are not exclusive, and they can be observed simultaneously in one lncRNA. The expression of the class of lncRNA encoded from enhancer regions is strictly connected with the class of the nearby mRNAs, allowing enhancers to physically associate with target promoters [42]. Finally, lncRNA can regulate allele-specific imprinting, allele-specific gene expression and inhibitory mechanisms [43,44,45].

## 3. Advanced Epigenetic Research in Human Stem Cells—A Novel Bioengineering Tool

Stem cells are undifferentiated cells with high proliferation capacity and capability of differentiating into a large variety of cell types in the body, and consequently show great promise in the field of regenerative medicine [46]. Specifically, stem cells can be grown in a laboratory and implanted into a patient to repair damaged tissues or organs. Stem cells can be categorized as either embryonic stem cells (ESCs), induced pluripotent stem cells (iPSCs), or adult/somatic stem cells (SSCs). ESCs show pluripotency and are responsible for differentiation of every cell type in the body during embryonic development [47]. iPSCs are engineered from adult cells to dedifferentiate into cells which behave similarly to ESCs and show pluripotency [48]. SSCs such as mesenchymal stem cells (MSCs) or hematopoietic stem cells (HSCs) are multipotent, found in developed tissues and organs, and important in repairing damaged tissue [49]. These cells show potential as tools in tissue engineering—following implantation to site of injury, they can stimulate a regenerative microenvironment via paracrine factors, which can both promote differentiation of resident precursor cells and provide an immunoregulatory function via modulation of T cells, mast cells, and macrophages [50,51].

Over time, with the help of chromatin-modifying enzymes, undifferentiated stem cells will establish a specific epigenetic profile to determine cell fate. In fact, these epigenetic modifications are responsible for ESC differentiation into various cell types during embryogenesis [52]. Upon lineage commitment, pluripotent factors are silenced and the expression of genes specific for the differentiated cell type increases. As stem cell application for therapy is limited by their efficacy in controlling cell fate and determination, manipulating epigenetic profiles via modifiers may be an important technique in regenerative medicine. These modifiers, including drugs, can reset gene-silencing effects, e.g., through DNA methylation or histone modifications [53]. 

Multiple layers of molecular control must be understood to elucidate the relevance of epigenetic changes in stem cell differentiation. Firstly, paracrine/juxtracrine signalling between cells can stimulate intracellular signalling pathways and cascades, activating or inactivating key transcription factors in cell fate regulation. Additionally, ncRNAs can inhibit mRNA translation of key genes. Methylation and histone modification profiles have been shown to differ significantly between ESCs and SSCs [54]. Deregulation of epigenetic modifications has also been implicated in various cancers and developmental disorders but remains poorly understood in the context of stem cells [55].

The epigenetic rearrangement of SSCs, marking commitment to a specific lineage, is of great importance in modern research. Further understanding of stem cell differentiation regulation could help to highlight potential therapeutic targets. Already in development, epigenetic modifier drugs may be able to reset/modify chromatin alterations and treat a variety of diseases, including cancers [56,57]. These drugs have been shown to enhance differentiation of stem cells when combined with existing procedures, i.e., culturing in differentiation media. However, epigenetic modification of stem cells by drug treatment alone is not sufficient to prompt differentiation [58]. Examples of relevant epigenetic modifiers include trichostatin A (TSA), a well-known and studied inhibitor of histone deacetylation, and 5-aza-deoxycytidine (5-aza-dC), an inhibitor of DNA methylation. A study conducted in 2007 showed that the treatment of murine bone marrow-derived mesenchymal stem cells (BMSCs) with either TSA or 5-aza-dC could enhance differentiation into neural-like cells, proved by the high expression of SRY-box transcription factor 2 (Sox 2), a marker for neural stem cells [59].

It is well established that MSCs are the common precursor of adipocytes and osteoblasts. In fact, the cell fate of MSCs is strictly balanced to maintain skeletal homeostasis and prevention of obesity. The ‘master regulators’ of osteogenesis and adipogenesis are Runt-related transcription factor 2 (RUNX2) and peroxisome proliferator-activated receptor γ (PPARγ). These key proteins are activated or inhibited by a complex array of signalling pathways, which may induce adipogenesis and inhibit osteogenesis or vice versa. These pathways include Wnt signalling, bone morphogenic proteins (BMPs), notch, hedgehog (HH) and fibroblast growth factors (FGFs) [60]. Additional epigenetic factors worth mentioning are methyltransferase enhancer of zeste homolog 2 (EZH2) and lysine demethylase 6A (KDM6A). EZH2 promotes adipogenesis and inhibits osteogenesis via the trimethylation of histone 3 at lysine 27 (H3K27me3), whereas KDM6A removes this methylation and opposes the action of EZH2. Experimentally, the overexpression of KDM6A in MSCs results in increased differentiation to osteogenic cell lines [61]. Both EZH2 and KDM6A may act upon key genes in cell fate determination of MSCs. For example, EZH2 is believed to methylate promoter regions, preventing the transcription of a number of genes, including Wnt genes, responsible for osteogenic differentiation through the actions of β-catenin via Wnt signalling cascades [61,62]. Furthermore, KDM6B, together with KDM4B, promotes osteogenic differentiation of human MSCs and is involved in the regulation of odontogenic differentiation of MSCs [62,63].

### 3.1. Gene Silencing through H3K27me by EZH2 and the PRC2 Complex

**Figure 1:**
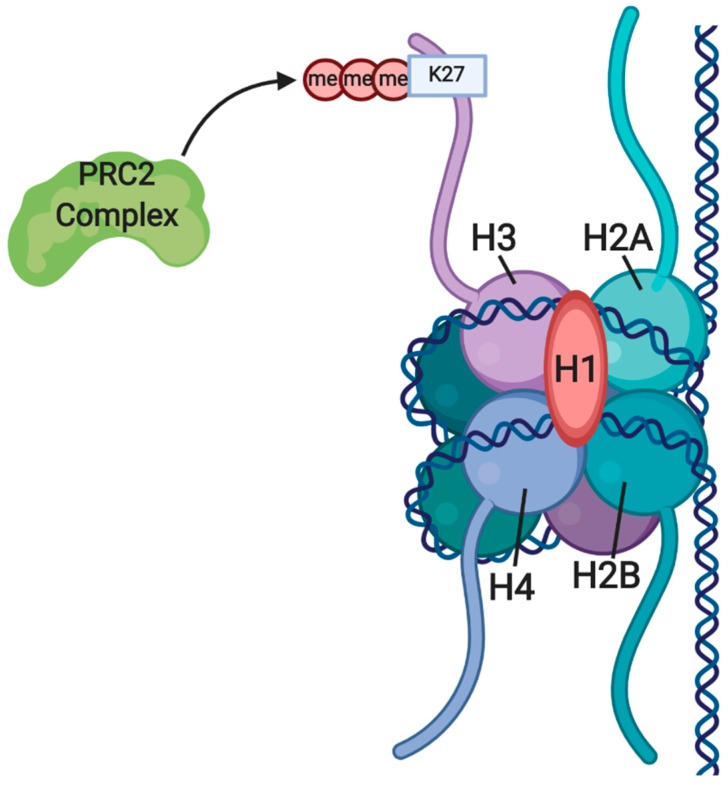
Structure of the nucleosome, with modification of histone tails. Histones bind together in an octamer structure to create the nucleosome around which the DNA strand wraps itself. Tail sections of the histones extend from the structure and are subject to post-translational modifications such as acetylation, methylation and phosphorylation. The repressive trimethylation mark of H3K27me3 and the PRC2 protein complex responsible for the modification are shown [1].

Together with protein regulator of cytokinesis (PRC) 1, an important activity of EZH2, a component of the protein complex PRC2, is reversible gene silencing by chromatin compaction. PRC2 is responsible for initiating gene silencing and PRC1 is believed to help maintaining this silencing (Figure 1) [64]. As well as trimethylation of histone H3K27me3 through the carboxy-terminal domain, EZH2 recruits DNA methyltransferases to the amino-terminal domain to further silence gene expression [65]. EZH2 maintains the multipotent phenotype of hematopoietic, neuron and muscle cell precursors, and is defined as a stem cell senescence-preventing gene, for its overexpression completely prevents senescence of HSCs [64]. Similarly, in neuron and muscle precursor cells, EZH2 plays an inhibitory role on genes specific for differentiation [66,67]. In undifferentiated myoblasts, EZH2 binds and represses miR-214. Moreover, EZH2 dissociation, followed by recruitment of MyoD and myogenin, sets the onset of skeletal muscle cell (SMC) differentiation. Upon activation of miR-214 transcription, this non-coding RNA binds and negatively regulates EZH2, further promoting SMC differentiation [65].

**Figure 2:**
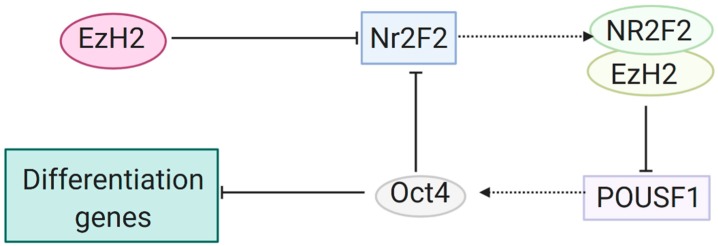
Maintenance of pluripotency in hESCs by EZH2 silencing of NR2F2. EZH2 silences the *Nr2f2* gene from transcribing via the trimethylation of histone 3 at lysine 27. EZH2 also binds with the gene product NR2F2 to repress transcription of the *Pou5f1* gene, which codes for OCT4. OCT4 prevents differentiation and maintains pluripotency of hESCs, and additionally acts to repress further transcription of *Nr2f2* [68,69].

Octamer-binding transcription factor 4 (OCT4), NANOG and SOX2 are key factors in maintenance of pluripotency in human embryonic stem cells (hESCs), as shown in Figure 2, and their regulation by the overlying epigenome is of great interest to researchers [68]. In 2017, Pursani et al. used microarray, quantitative reverse transcription PCR (RT-qPCR) and chromatin immunoprecipitation (ChIP) techniques to identify the roles of EZH2 and the nuclear receptor subfamily 2 group f member 2 (NR2F2) transcription factor in the expression of the octamer-binding transcription factor 4 (OCT4) coding the POU class 5 homeobox 1 (*Pou5f1*) gene during differentiation of hESCs [69]. During differentiation of cardiac precursors and cardiomyocytes, NR2F2 was upregulated, while EZH2 was downregulated. ChIP analyses revealed that EZH2 silences Nr2f2 expression by trimethylation of H3K27me3 at the promoter region in undifferentiated cells. Similarly, EZH2 represses Pou5f1 in differentiated but not in undifferentiated cells. Moreover, the undifferentiated state is preserved through the repression of Nr2f2 by OCT4, one of the key transcription factors involved in maintaining pluripotency in cells [68]. ChiP analyses show interactions between Nr2f2 and both EZH2 and Pou5f1, leading to the conclusion that NR2F2 recruits EZH2 to bind at the Pou5f1 promoter to silence the expression of OCT4 during cardiac differentiation of hESCs [68,69].

**Figure 3:**
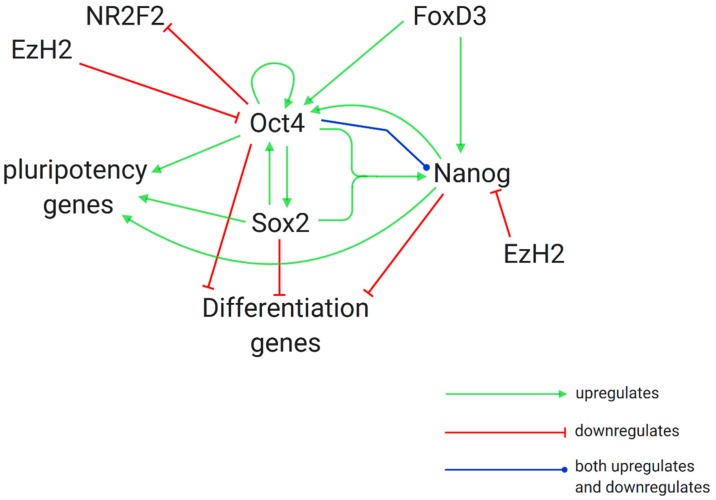
Maintenance of pluripotency vs. differentiation in hESCs and the role of the histone modifier EZH2. *OCT4*, *SOX2* and *NAOG*, key genes in maintenance of pluripotency in hESCs, form a complex interdependent regulatory network. The histone modifier EZH2 disturbs the balance of this regulatory circuit and thereby triggers differentiation by silencing transcription of the genes which translate the OCT4 and NANOG products [72,73].

It is well known that NANOG levels in stem cells positively correlate with self-renewal capacity and pluripotency maintenance. Using quantitative immunofluorescence and cytometry techniques, Villasante et al. studied levels of NANOG in both wild-type and EZH2-deficient iPSCs. High levels of NANOG were significantly linked to low levels of EZH2, while low levels of NANOG were significantly linked to high levels of EZH2. ChIP analysis showed interactions between EZH2, and thereby its silencing mark H3K27Me3, and the promoter of NANOG, confirming that EZH2 is a negative regulator of NANOG, binding directly to its promoter (Figure 3) [70].

SOX2 and OCT4 expressions are essential to iPSC induction, and SOX2 is believed to be a key player in pluripotency maintenance. Studies indicate that SOX2 and OCT4 positively influence each other expression and cooperate in the downstream regulation of NANOG expression. In this way, a complex interdependent network between these master regulators maintains stem cell phenotype (Figure 3) [71].

### 3.2. Further Research on Epigenetic Modifications and Epigenetic Inheritance

The trimethylation of histone H3 lysine 4 (H3K4me3) at transcriptional start sites recruits remodelling enzymes and histone acetylases and upregulating transcription, while the negative regulator H3K27me3 behaves oppositely. These two histone modifiers act together on developmental genes to create ‘bivalent domains’ of the genome [74,75]. The genes important for differentiation, that are both up- and downregulated via opposing modifications of the associated histones, are believed to be silenced, but kept in a ‘poised’ state, ready for activation upon the appropriate cues. A study on the mouse genome linked almost half of all bivalent domains to binding sites of the key transcription factors SOX2, OCT4 and/or NANOG [74]. Notably, most bivalent domains eventually resolve into areas marked by either H3K4me3 or H3K27me3, depending on lineage specificity.

Stem cells play a key role in furthering bioengineering technologies and, consequently, detailed understanding of cell fate regulation is paramount to developing treatments. EZH2 prevents differentiation of neural, muscle, bone and hematopoietic precursor cells of MSCs. However, EZH2 is also known to inhibit the activity of pluripotency genes such as NANOG and Pou5f1 in ESCs, suggesting that histone modifiers are cell and phase specific, playing different roles depending on cell type and state of differentiation.

As well as the histone modifiers described above, DNA methyltransferases play a role in stem cell differentiation [76]. This family of enzymes catalyse the methylation of CpG islands in DNA, a process responsible for many functions, including repression of transcription at promoters. The methylation profile is maintained during mitosis, where the DNA methyltransferase DNMT1 is responsible for copying methylated DNA patterns from the template strand to the new strand. However, the mechanism of histone modification inheritance is not well understood and remains a topic of research [77]. Nevertheless, it has been suggested that histone modifications are preserved throughout DNA replication via histone recycling, and not via the establishment of patterns on new component histones [78]. Evidence from studies on the mechanisms underlying epigenetic regulation and chromatin states in yeast and other organisms suggest a conservative or semi-conservative model of nucleosome assembly and subsequent epigenetic inheritance, providing a greater mechanistic understanding of heterochromatin preservation [79]. Epigenetic modifier drugs can be used to reset DNA methylation and histone modification profiles, breaking the inheritance pattern and thereby allowing manipulation of cell fate for therapeutic purposes, mostly due to their tumour-specific anti-cancer activities [56]. For example, the first drug developed for the treatment of T-Cell Lymphoma was Vorinostat (SAHA, Zolina), which, being structurally similar to TSA, is associated with HDAC inhibition. Romidepsin and Belinostat are other drugs with HDAC inhibitory activity that are widely used in clinical trials [80,81].

### 3.3. Cancer Research and Anti-Cancer Therapy—Living in the Shadow of Epigenetic Genome Modification

Heritable changes in gene function, including epigenetic regulatory mechanisms such as DNA methylation and histone modification, play an important role in cancer progression [82]. Recently, epigenetic modifications were highlighted as a novel and significant hallmark for assaying various types of cancer. Acetylation, methylation and demethylation of either DNA or histones, long non-coding RNAs or nucleosome remodelling are key epigenetic mechanisms that do not involve mutations nor spontaneous changes in the nucleotide sequences of double-stranded DNA [83]. Carcinogenesis depends not only on aggregation of point mutations and mismatches within genetic codes, but also on aberrant epigenetic regulations. Cancer prognosis is therefore related to maintaining genomic stability and spatially controlling gene expression [84]. Hence, targeting epigenetic alterations shows promise for dissemination of tumourigenesis, tumour progression, tumour metastasis and therapeutic resistance to chemotherapy [85]. Epigenetic modifications are not the only targets for cancer therapies, as they work in concert with mechanisms controlling transcriptional activity of malignant cells and therefore are considered as a whole [86]. Specifically, early onset detection of three of the most frequent cancers in men—colorectal, prostate and lung—is possible through non-invasive identification of aberrant DNA methylation at the promoter region of oncogenes and tumour-suppressor genes [87]. Analogically, cell-free DNA methylation-based tests are currently being developed for early onset detection of equivalent common cancer in women—colorectal, lung and breast cancers [88]. Genome-wide association studies (GWASs) enabled description of heritable epigenetic mechanisms, their effect on signalling pathways and reversible alterations in various types of cancers, currently recognized as their hallmarks [89]. Cis-acting elements, called enhancers, are identified as hallmarks for the identification of cancer-related putative enhancers when associated with the modulation of histones of multiple transcription factor binding sites upregulating transcription [90]. Recently, regulating gene expression by reacetylation of histones, histone deacetylases inhibitors were highlighted as potential therapeutic agents. By opposing the carcinogenesis effect of histone deacetylation, these inhibitors are involved in cancer initiation and development, through cell cycle arrest and induced apoptosis of cancerous cells [91]. Interestingly, epigenetic processes not only aid the diagnosis and prognosis of disease pathogenesis, but they provide useful information on the role of epigenetics in responsiveness to drugs [92]. For instance, the extract of a traditional Chinese herbal compound, known as rocaglamide, reduces chemotherapy mediated toxicities. Simultaneously, rocaglamide protects from chemotherapy side effects such as DNA damage-induced toxicity by halting DNA upregulation of p53 transcription factor [93]. An epigenome-wide study, conducted under the gingiva-buccal oral squamous cell carcinoma, identified disease-related dysregulation of promoter methylation and the expression of transcription factors to be repaired by epigenetic modification, including, respectively, upregulation and downregulation of DNMT3B and ten-eleven translocation methylcytosine dioxygenase (TET1) [94]. Studies conducted on triple-negative breast cancer confirmed that aberrant methylation of DNA contributes to silencing of tumour-related genes, leading to malignant transformations of healthy cell lines [95]. Other clinical studies on RNA–RNA interactions underlying the mechanism of breast cancer identified upregulation of human receptor tyrosine-protein kinase erbB-2 (ERBB2) by BCLIN25 through regulation of promoter CpG methylation on miR-125ERBB2 [96]. Methylation levels in adenomatous polyposis coli (*APC*), homeobox gene A9 (*HOXA9*), retinoic acid receptor beta 2 (*RARβ2*)*,* and Ras association domain-containing protein 1 (*RASSF1A*) were assessed as a novel non-invasive method for lung cancer subtyping. Higher methylation patterns of *HOXA9* and *RASSF1A* were detected in small-cell lung cancer rather than non-small-cell lung cancer [97]. Quantitative panel assessment of two-gene promoter methylation patterns (singleplex-miR-34b/c, miR-193b and multiplex-APC, GSTP1, RARβ2, respectively), in tissue biopsies and urine of prostate cancer patients demonstrated higher methylation levels of both panels compared to controls [98]. Analogically to epigenetic changes, such as methylation of histones and DNA, RNA modifications are useful in the identification and progress of diseases, including tumours and cancer. Current research on RNA modifications describes a further function of the most commonly modified RNA, m^6^A RNA, such that N6-methyladenosine modification of m^6^A RNA demonstrated a role in gastrointestinal tract cancers [99]. m^6^A RNA has also been confirmed to modulate gene expression in clear-cell renal-cell carcinoma (ccRCC), identified as either hypermethylated or hypomethylated m^6^A peaks in a m^6^A transcriptome-wide map of human ccRCC [100]. The epigenetic reader of the N6-methyladenosine modification of m^6^A RNA, called YTH N6-methyladenosine RNA binding protein 1 (YTHDF1), was further characterized and distinguished by its overexpression in Merkel cell carcinoma, indicating its involvement in the Merkel cell polyoavirus (*MCPyV*) gene expression [101]. Another study found that *miR-506* targets the epigenetic factor ubiquitin-like with PHD and ring finger domains 1 (*UHRF1*) and inhibits colorectal cancer via the KiSS-1 metastasis suppressor (KISS1)–phosphoinositide 3-kinases (PI3K)–nuclear factor kappa-light-chain-enhancer of activated B cells (NF-κB) signalling pathway [102]. Pan-cancer analysis, including the integrative altered methylation studies on *DNMT3B* methylation patterns, revealed key metabolic genes, such as solute carrier family 2 member 1 (*SLC2A1*)*,* ATP citrate synthase (*ACLY*) and lysine acetyltransferase 2A (*KAT2A*), as commonly contributing factors to errors of histone modifications and DNA methylation [103].

Even though the contribution of epigenetics to prognosis and diagnosis of neurodegenerative diseases is well established, epigenetic modifications have also demonstrated a prominent role in the metastasis of various types of cancer. Consequently, apart from mito-epigenetic changes in mitochondrial RNAs (mtRNA), including mt-mRNA, mt-tRNAs and mt-rRNAs (mtRNAs), and mitochondrial DNAs (mtDNA) controlling replication, transcription and translation of mitochondrial genes, hydroxyl methylation of circular mitochondrial DNA, mtDNA and nDNA-derived non-coding RNAs appears to play a vital role in carcinogenesis pathways, due to the role of mitochondrial dysfunction in cancers [104]. Studies focussing on the methylation of Alu repeats in areas of repressed chromatin compared normal and cancer tissues associating Alu repeats with the digestive organ expansion factor homolog (*diexf*) gene promoter region. Due to its hypomethylated state in various cancers, *diexf* was identifyied as a potential tumoral biomarker [105]. In comparison, the hypermetabolic state, described as encouraging modifications in cellular epigenetics, plays a vital role in transcriptional silencing of retrotransposon elements through upregulation of DNA methyltransferases, leading to hypermethylation of DNA [106]. Recently, Twist family BHLH transcription factor 2 (Twist2) was found to promote changes in the binding pattern of myoblast determination protein 1 (MyoD), causing it to bind oncogenes. As Twist2 directs chromatin closing at the myogenic locus, this process simultaneously directs chromatin opening at the oncogenic locus [107]. Therefore, Twist2 is a potential target for the therapeutic treatment of rhabdomyosarcoma (RMS), a cancer forming from myoblast-like cells.

## 4. Epigenetic Genome Modification and Regenerative Medicine

In recent years, regenerative medicine largely benefitted from advances in identifying novel and common causal cancer risk variants using genome-wide association studies. Researchers conducting large-scale sequencing studies identified multiple somatic mutations in epigenome organization and the crucial roles of epigenome modifications in carcinogenesis. As an example, a combination of in silico genomic feature annotations with association analysis, including linkage disequilibrium, genetic association and enriched genomic features, known as a Bayesian approach, described more than 200 breast cancer-related signals [108]. The regenerative potential of transient, genome-wide epigenomic remodelling was recently described in the process of organoid formation and liver regeneration following tissue damage [109]. Epigenetic genome modifications are not only considered in studies concerning the regeneration of tissue and stem cell, but most importantly in studies concerning the prognosis and metastasis of various types of cancers, specifically in relation to tumour microenvironment, immune regulation, tissue-level physical forces and other cell-intrinsic mechanisms, including integration of transcriptomics and metabolomics [110]. In-depth study of the protective activities associated with the therapeutic potential of natural products, including spices, teas and plants, enabled the identification of key epigenetic alterations and signalling pathways by targeting specific transcription factors [111]. In fact, natural plant products showed immunomodulatory activity acting as anti-oxidant and anti-tumour agents—for example, *Viscum album* L. extracts were observed to downregulate TGF-β, providing clinical benefits to subjects affected by invasive tumours [112]. Moreover, clues concerning epigenetic mechanisms, with potential in regenerative therapies, are partially drawn from epigenetic reprogramming processes required for the development of an embryo from fertilized egg and the establishment of the totipotency of cells in vivo [113]. Nonetheless, detailed epigenomic profiling of the human body allowed researchers to identify epigenetic effects on disease development, including regions of nucleosome-free DNA targeted by regulatory factors [114]. As an example, mutations in methyl-CpG binding protein 2 (MeCP2), leading to Rett syndrome, cause genome-wide amplification of histone acetylation, promoting the investigation into the association of the chromatin environment of MeCP2 target genes and the density of histones H1, H2B and H3 [115]. Recent studies on Parkinson’s disease (PD) identified a global DNA hypermethylation in monogenic leucine-rich repeat kinase 2 (LRRK2)-associated PD patients, as well as in sporadic PD patients, as another approach for diagnosing and targeting the neurodegenerative disease, in addition to already established hallmarks following the loss of midbrain dopaminergic neurons [116]. Profiling of transcriptome-wide N6-methyladenosine sites, in the context of fragile X syndrome, led to identification of fragile X mental retardation protein (FMRP) as a potential target of m^6^A [117]. Furthermore, proteins downregulated by FMRP demonstrated not only increased m^6^A sites, but also interactions of FMRP with m^6^A reader YTHDF2, suggesting a possible regulation of m^6^A-marked mRNA by FMRP. Additionally, considering technical ways for quantifying and qualifying epigenetic modifications of DNA and chromatin in the context of regenerative medicine, epigenetic profiling is accessible and cost effective thanks to chromatin immunoprecipitation assays associated with μChIP-PCR or microarrays [118]. 

## 5. Epigenome and Human Clinical Trials

Multiple studies and clinical trials on epigenetic drugs, listed in Table 1 and Table 2, aim at improving their effectiveness and safety for cancer treatment, as well as achieving a better understanding of the regulatory roles that epigenetic modifications play in cancer and, especially, cancer stem cells. Furthermore, several studies researching the influence of epigenetic modifications in regenerative medicine are also presented. 

### 5.1. Epigenetic Drugs in Cancer

The 5-azacytidine (azacitidine) and 5-aza-2’-deoxyctidine (decitabine) azanucleosides were the subject of multiple epigenetics-related clinical trials. While azacitidine was first approved for the treatment of myelodysplastic syndrome (MDS), these compounds are now commonly used in the treatment of acute myeloid leukaemia (AML) [137,138]. The chemical structure of azacitidine is similar to both DNA methyltransferase inhibitor and hypomethylating agent. Hence, it can be used to downregulate methylation [139]. Its epigenetic activity has shown to silence tumour suppressor genes [140]. Furthermore, azanucleosides improve both the differentiation and the proliferation of normal cells [138]. 

Clinical trial NCT02993523 investigates whether the combination of azacitidine and a newly developed drug called venetoclax will improve the latter’s performance in AML treatment. In addition to the evaluation of effectiveness of venetoclax, the eventual variation caused by genetic factors and drug retention will also be investigated [128]. In turn, clinical trial NCT03164057 investigates whether epigenetic priming, in combination with DNA methyltransferase inhibitor (such as azacitidine), can improve the effectiveness of chemotherapy in the treatment of MDS or AML. Once the clinical trial is over, the tolerance of the patients to chemotherapy and epi-drug treatment will be evaluated in addition to changes in the methylation profile of the leukaemic cells [129]. Furthermore, clinical trial NCT01928576 investigates priming epigenetic drug treatment in various non-small-cell lung cancer (NSCLC) [135]. The treatment begins with a low dose of nivolumab (3 mg/kg/2 wk), a commonly used immune checkpoint modulator, followed by epigenetic priming. Assessment of the progress will then be carried out using RECIST 1.1 criteria to establish the effectiveness of the treatment [141]. Azacitdine was also tested in a prostate cancer treatment clinical trial. The treatment was successful when and if the prostate-specific antigen (PSA) response rate was increased 2-fold after at least 3 months of treatment. In total, 34 patients were analysed, and the success rate was 55.8%, suggesting that the drug is relatively safe for use, with no apparent side effects, apart from singular cases of nausea and vommiting [127]. 

Due to the toxic effects of high doses of azanucleosides, hydralazine and valproate were used as an alternative in another trial [142]. These compounds show the ability to reverse methylation and deacetylation, as well as restore the activity of tumour suppressor genes [143]. In clinical trial NCT00404508, these inhibitors were used in an attempt to overcome tumour chemotherapy resistance [119]. Epigenetic modifications, methylation and histone deacetylation were analysed following the treatment. In the published results, 4/5 patients who finished the trial either saw an improvement in their treatment or reached stabilization. Therefore, the epi-drugs were considered effective in ensuring the success of chemotherapy [144]. In clinical trial NCT00404326, hydralazine and valproate were also studied in combination with cisplatin-based chemotherapy [122]. The results described by Cruz-Hernández et al. stated that the drug affects oncogene expression. However, 72.7% patients did not exhibit a significant effect. The clinical trial would need to be repeated with a higher number of patients to achieve a significant conclusion [145]. In turn, fludarabine is being used in clinical trial NCT02497404 following a five-day azacitidine treatment as a less-intensive methylation inhibitor. This trial includes patients in remission with a high-risk of myeloid malignancies receiving hematopoietic stem cells from human leukocyte antigen-matched donors. The results of the study will offer insights into the combination of azacitidine priming with hematopoietic stem cell transplants and their possible role in cancer remission and maintenance of normal cell function [130,146].

Azacitidine is being investigated in clinical trial NCT01566695 as a supplementation for red blood cell transfusion in the treatment of MDS [136]. Progression of MDS is associated with risk of AML, with the treatment of the former considered as a preventive measure for the latter [147]. For the purpose of the study, participants will be administered azacitidine or placebo daily for the first three weeks of their four-week blood transfusion treatment cycle. The patients will then be observed for a minimum of 56 days, the red blood cell transfusion cut-off point, and the effectiveness of the treatment for the two groups will be statistically analysed considering patient disease recovery and mortality [148].

The safety and effectiveness of azacitidine for the treatment of MDS was also investigated in clinical trial NCT01201811 [125]. Firstly, the participants were screened for 7 days, four weeks before taking azacitidine (75 mg/m^2^), to clarify their MDS diagnosis and condition before the drug trial. The patients took azacitidine until the end of the hematopoietic blood transfusion treatment. The trial resulted in partial progress after treatment for 75% of the participants and proved that the dose of azacitidine was safe [149].

A phase I clinical trial investigated the use of an anti-sense oligonucleotide to DNMT1, called MG98, testing its safety and toxicology. As DNMT1 enhances tumour growth, its anti-sense oligonucleotide was designed to oppose this activity. The participants were treated for solid tumours by increasing the doses of the drug, administering it continuously and intravenously for a week (700 mg/m^2^/wk dose). No significant difference in the suppression of DNMT1 activity was observed between the effects of low and high doses of MG98. Overall, the small range of doses of MG98 used in the study was deemed to be safe for future trials [123].

### 5.2. Mechanisms in Cancer

One of the studies aimed to identify the actions of histone 3 lysine 9 dimethylation (H3K9me2) and the G9a enzyme (histone-lysine N-methyltransferase, H3 lysine 9 specific 3) on glioma cancer stem cells. CD133-positive cells were observed to be H3K9me2 negative, although most cancer cells exhibit this modification. By overexpressing G9a, its frequency was increased, affecting the expression of CD133 and Sox2 promoter regions, both involved in cell self-renewal. The results suggested that G9a-dependent H3K9me2 regulates self-renewal in cancer stem cells, since it is involved in preserving the undifferentiated state of stem cells [121]. Moreover, in a study by Torres et al., chromatin expression was also observed to be involved in maintaining the undifferentiated state of cancer stem cells, with the linker histone H1.0 identified as a cancer stem cell marker. Furthermore, silencing the H1F0 increased self-renewal activity in cancer stem cells, showing its involvement in regulatory mechanisms [126]. 

Furthermore, in a trial conducted by van den Boom et al., self-renewal and undifferentiated characteristics of human leukaemic stem cells from AML patients were observed to be strongly regulated by epigenetic modifications via polycomb protein PRC1.1. Cell growth decreased when PRC1.1 complex protein (KDM2B, PCGF1, and BRCOR(L1)) was knocked out. The complex was found to be influenced by histone modifier H327me3 PRC2, as the latter’s absence coincided with the onset of PRC1.1 transcription. However, in the presence of H327me3, chromatin enhances the polycomb protein. Evidence revealed that the complex interacts with key genes involved in leukaemogenesis, including PKM and LDHA, independently of H327me3. This would suggest that epi-drugs could potentially manipulate cancer stem cell maturation. In conclusion, PRC1.1 complex is essential for the maturation of leukaemic stem cells and has potential in cancer treatment [124].

The role of tyrosine kinase inhibitor (TKI)-resisting chronic myeloid leukaemia (CML) cells in treatment failures will be investigated in clinical trial NCT03481868. TKIs are effective in the treatment of CML and are used to target the BCR-ABL chimeric protein found in leukaemic stem cells. The clinical trial aims to identify the reason for cancer remission after treatment and to explain TKI resistance in CML leukaemic stem cells, leading to an understanding of the mechanisms underlying the epigenetic modifications that take place in the surviving CML cells. So far, the distinct DNA methylation profiles of CD34-CD15 + of CML before treatment were analysed and compared to the profile of healthy donors. Biomarkers deregulating the methylation were identified in the two target gene populations, monitoring changes in the mediators involved [133]. 

It has been hypothesized that epigenetic modifications affect biological aging and therefore age-related deterioration or diseases such as Alzheimer’s Disease [150]. To investigate this further, clinical trial NCT03871296 aims to identify biological ageing acetylation markers in patients undergoing allogenic hematopoietic stem cell transplantations and adjust the age criteria for patients to undergo cancer treatments, such as hematopoietic stem cell transplants [132]. So far, the group has published results related to Down Syndrome patients, comparing the methylation patterns related to trisomy of chromosome 21 to familial controls, concluding that the abnormal genetic modification expression does accelerate biological aging [151].

### 5.3. Regenerative Medicine

The effect of epigenetic modifications on wound healing, specifically the activity and changes in expression driven by methylation of fibroblast growth factor 2 (FGF2), were investigated in clinical trial NCT01663298. Additionally, the clinical trial hoped to analyse changes in the expression of FGF2 caused by environmental factors, specifically smoking and diabetes. To assess FGF2 methylation expression, periodontal tissue after implant surgery was studied. By achieving a better understanding of the mechanisms underlying wound healing, future results of this trial may lead to more effective proliferation and differentiation of stem cells for regenerative medicine [120]. The roles of epigenetics in wound healing are also being studied in clinical trial NCT03793062, which aims to compare the epigenetic and transcriptional changes between healing and non-healing tissue (chronic wounds). Epigenetic expression may regulate tissue healing, with identification of the difference between the epigenetic profiles of the two tissues possibly enabling advances in regenerative medicine [134].

Clinical trial NCT04122742 aims to characterize specific acetylation markers in Rubinstein–Taybi syndrome (RSTS), using the disease as an epigenetic neurodevelopmental model. The two causative genes of the syndrome are CREBBP and EP300, encoding CBP and p300, respectively, which have direct roles in chromatin remodelling and transcriptional co-activation. The study will investigate the epigenetic character of the disease through the analysis of CBP-dependent histone markers in primary fibroblast cultures harvested from skin biopsies and blood samples of RSTS patients. Induced pluripotent stem cells (iPSCs) will be generated from the fibroblast cultures. The results could contribute to the understanding of how acetylation influences neuronal stem cell differentiation by expressing CREBBP [131].

## 6. Conclusions

Epigenetic studies will most likely be the basis of future cancer therapies, as this mechanism plays major roles in tumour formation, malignancy and metastasis. Hence, there is a large number of currently designed or tested clinical approaches, based on compounds regulating the epigenetic pathways in various types of tumours. Furthermore, the same principles apply to stem cells, and it is likely that research and application of epigenetic studies will allow for further development of approaches associated with stem cell bioengineering. 

## Figures and Tables

**Table 1:** A list of completed research studies and clinical trials relating to the topic, arranged in alphabetical order.

	Completed Research Studies and Clinical Trials				
No.	Study Title	Condition(s)	Intervention(s)	Reference	ClinicalTrials.gov Identifier
1.	A Phase II Study of Epigenetic Therapy to Overcome Chemotherapy Resistance in Refractory Solid Tumors	Refractory solid tumours	Hydralazine and magnesium valproate	[119]	NCT00404508
2.	Gene Expression Variation and Implant Wound Healing among Smokers and Diabetics	- Smoking - Diabetes	Dental implant surgery	[120]	NCT01663298
3.	Histone Methyltransferase G9a and H3K9 Dimethylation Inhibit the Self-Renewal of Glioma Cancer Stem Cells	Glioma	Histone methyltransferase G9a and H3K9 dimethylation	[121]	N/A
4.	Hydralazine and Valproate Plus Cisplatin Chemoradiation in Cervical Cancer	Cervical cancer	Hydralazine and magnesium valproate	[122]	NCT00404326
5.	Phase I Study of MG98, an Oligonucleotide Antisense Inhibitor of Human DNA Methyltransferase 1, Given as a 7-Day Infusion in Patients with Advanced Solid Tumors	Cancer	MG98, an oligonucleotide to DNA DNMT1	[123]	N/A
6.	Non-Canonical PRC1.1 Targets Active Genes Independent of H3K27me3 and Is Essential for Leukemogenesis	Acute myeloid leukaemia (AML)	Downregulation of non-canonical PRC1.1 complex delays or prevents both carcinogenesis and its development in mice models	[124]	N/A
7.	Study of Azacitidine in Adult Taiwanese Subjects with Higher-Risk Myelodysplastic Syndromes (MDS)	Myelodysplastic syndromes	Azacitidine	[125]	NCT01201811
8.	The Linker Histone H1.0 Generates Epigenetic and Functional Intratumor Heterogeneity	- Breast cancer - Glioma and glioblastoma - Melanoma - Kidney renal papillary cell carcinoma Liver cancer	Reversible-silencing of linker histone H1.0 to manipulate tumour proliferation	[126]	N/A
9.	Vidaza to Restore Hormone Thx Prostate	Prostate cancer	Azacitdine for injectable suspension	[127]	NCT00384839

**Table 2:** A list of ongoing research studies and clinical trials relating to the topic, arranged in alphabetical order.

	Ongoing Research Studies and Clinical Trials				
No	Study Title	Condition(s)	Intervention(s)	Reference	ClinicalTrials.gov Identifier
1.	A Study of Venetoclax in Combination with Azacitidine versus Azacitidine in Treatment Naïve Subjects with Acute Myeloid Leukemia Who Are Ineligible for Standard Induction Therapy	Acute myeloid leukaemia (AML)	- Azacitidine - Venetoclax - Placebo	[128]	NCT02993523
2.	A Trial of Epigenetic Priming in Patients with Newly Diagnosed Acute Myeloid Leukemia	- Acute myeloid leukaemia - Myelodysplastic syndromes	- Azacitidine - Decitabine - Cytarabine - Stem cell transplant - etc.	[129]	NCT03164057
3.	Azacytidine Prior to in Vivo T-cell Depleted Allo Stem Cell Transplant for Patients with Myeloid Malignancies in CR	- Leukaemia - Erythroblastic - Acute myelodysplastic syndromes	- Azacitidine - Fludarabine - Melphalan - Alemtuzumab	[130]	NCT02497404
4.	Diagnosis of RSTS: Identification of the Acetylation Profiles as Epigenetic Markers for Assessing Causality of CREBBP Variants	Rubinstein–Taybi syndrome	To investigate: - Generation of induced pluripotent Stem cells (iPSC) from fibroblasts obtained by skin biopsy - Histone acetylation profiles of cells of SRT patients with CREBBP mutations - Functional involvement of identified epigenetic alterations - Culture of lymphoblastoid line from blood sample	[131]	NCT04122742
5.	DNA Methylation in Allogenic Hematopoietic Stem Cell Transplantation	- Aging - Stem cell transplant complications	Investigation comparing DNA methylation of patients	[132]	NCT03871296
6.	EPIgenetics and in Vivo Resistance of Chronic Myeloid Leukemia Stem Cells to Tyrosine Kinase Inhibitors (EPIK)	- Chronic myeloid leukaemia (CML) - Chronic Phase	Collection of blood and bone marrow	[133]	NCT03481868
7.	Genetic and Epigenetic Basis of Chronic Wounds	Chronic wounds	Observational	[134]	NCT03793062
8.	Phase II Anti-PD1 Epigenetic Therapy Study in NSCLC	Non-small-cell lung cancer, epigenetic therapy	- Azacitidine - Entinostat - Nivolumab	[135]	NCT01928576
9.	The Efficacy and Safety of Oral Azacitidine Plus Best Supportive Care versus Placebo and Best Supportive Care in Subjects with Red Blood Cell (RBC) Transfusion-Dependent Anemia and Thrombocytopenia Due to International Prognostic Scoring System (IPSS) Low Risk Myelodysplastic Syndrome (MDS)	Myelodysplastic syndrome	- Oral azacitidine - Placebo	[136]	NCT01566695

## References

[1] Crick F. (1970). Central dogma of molecular biology. Nature.

[2] Holliday R. (1987). The inheritance of epigenetic defects. Science.

[3] Egger G., Liang G., Aparicio A., Jones P.A. (2004). Epigenetics in human disease and prospects for epigenetic therapy. Nature.

[4] Lopomo A., Burgio E., Migliore L. (2016). Epigenetics of Obesity. Prog. Mol. Biol. Transl. Sci..

[5] Muka T., Koromani F., Portilla E., O’Connor A., Bramer W.M., Troup J., Chowdhury R., Dehghan A., Franco O.H. (2016). The role of epigenetic modifications in cardiovascular disease: A systematic review. Int. J. Cardiol..

[6] Lovrei L., Maver A., Zadel M., Peterli B. (2013). The Role of Epigenetics in Neurodegenerative Diseases. Neurodegener. Dis..

[7] Carson C., Lawson H.A. (2018). Epigenetics of metabolic syndrome. Physiol. Genom..

[8] Huang T., Peng X., Li Z., Zhou Q., Huang S., Wang Y., Li J., Song Y. (2018). Epigenetics and bone diseases. Genet. Res. (Camb.).

[9] Del Real A., Riancho-Zarrabeitia L., López-Delgado L., Riancho J.A. (2018). Epigenetics of Skeletal Diseases. Curr. Osteoporos. Rep..

[10] Okano M., Bell D.W., Haber D.A., Li E. (1999). DNA methyltransferases Dnmt3a and Dnmt3b are essential for de novo methylation and mammalian development. Cell.

[11] Tabolacci E., Pietrobono R., Moscato U., Oostra B.A., Chiurazzi P., Neri G. (2005). Differential epigenetic modifications in the FMR1 gene of the fragile X syndrome after reactivating pharmacological treatments. Eur. J. Hum. Genet..

[12] Feinberg A.P., Tycko B. (2004). The history of cancer epigenetics. Nat. Rev. Cancer.

[13] Adenocarcinoma D., Sato N., Maitra A., Fukushima N., Heek N.T., Van Matsubayashi H., Iacobuzio-Donahue C.A., Rosty C., Goggins M., van Heek N.T. (2003). Frequent hypomethylation of multiple genes overexpressed in pancreatic ductal adenocarcinoma. Cancer Res..

[14] Cichowski K., Shih T.S., Schmitt E., Santiago S., Reilly K., McLaughlin M.E., Bronson R.T., Jacks T. (1999). Mouse models of tumor development in neurofibromatosis type 1. Science.

[15] Sakai T., Toguchida J., Ohtani N., Yandell D.W., Rapaport J.M., Dryja T.P. (1991). Allele-specific hypermethylation of the retinoblastoma tumor-suppressor gene. Am. J. Hum. Genet..

[16] Schneider E., Pliushch G., Hajj N., El Galetzka D., Puhl A., Schorsch M., Frauenknecht K., Riepert T., Tresch A., Müller A.M. (2010). Spatial, temporal and interindividual epigenetic variation of functionally important DNA methylation patterns. Nucleic Acids Res..

[17] Holliday R., Pugh J.E. (1975). DNA modification mechanisms and gene activity during development. Science.

[18] Chen C., Yang M.C.K., Yang T.P. (2001). Evidence that silencing of the HPRT promoter by DNA methylation is mediated by critical CpG sites. J. Biol. Chem..

[19] Fatemi M., Hermann A., Gowher H., Jeltsch A. (2002). Dnmt3a and Dnmt1 functionally cooperate during de novo methylation of DNA. Eur. J. Biochem..

[20] Siegfried Z., Eden S., Mendelsohn M., Feng X., Tsuberi B.Z., Cedar H. (1999). DNA methylation represses transcription in vivo. Nat. Genet..

[21] Wolf S.F., Jolly D.J., Lunnen K.D., Friedmann T., Migeon B.R. (1984). Methylation of the hypoxanthine phosphoribosyltransferase locus on the human X chromosome: Implications for X chromosome inactivation. Proc. Natl. Acad. Sci. USA.

[22] Yang A.S., Estécio M.R.H., Doshi K., Kondo Y., Tajara E.H., Issa J.-P.J. (2004). A simple method for estimating global DNA methylation using bisulfite PCR of repetitive DNA elements. Nucleic Acids Res..

[23] Weber M., Davies J.J., Wittig D., Oakeley E.J., Haase M., Lam W.L., Schübeler D. (2005). Chromosome-wide and promoter-specific analyses identify sites of differential DNA methylation in normal and transformed human cells. Nat. Genet..

[24] Herman J.G., Graff J.R., Myöhänen S., Nelkin B.D., Baylin S.B. (1996). Methylation-specific PCR: A novel PCR assay for methylation status of CpG islands. Proc. Natl. Acad. Sci. USA.

[25] Lennartsson A., Ekwall K. (2009). Histone modification patterns and epigenetic codes. Biochim. Biophys. Acta Gen. Subj..

[26] Zhao J. (2007). Sumoylation regulates diverse biological processes. Cell. Mol. Life Sci..

[27] Cedar H., Bergman Y. (2009). Linking DNA methylation and histone modification: Patterns and paradigms. Nat. Rev. Genet..

[28] Turner B.M. (2007). Defining an epigenetic code. Nat. Cell Biol..

[29] Strahl B.D., Allis C.D. (2000). The language of covalent histone modifications. Nature.

[30] Li F., Huarte M., Zaratiegui M., Vaughn M.W., Shi Y., Martienssen R., Cande W.Z. (2008). Lid2 Is Required for Coordinating H3K4 and H3K9 Methylation of Heterochromatin and Euchromatin. Cell.

[31] Lee J.S., Shukla A., Schneider J., Swanson S.K., Washburn M.P., Florens L., Bhaumik S.R., Shilatifard A. (2007). Histone Crosstalk between H2B Monoubiquitination and H3 Methylation Mediated by COMPASS. Cell.

[32] Duncan E.M., Muratore-Schroeder T.L., Cook R.G., Garcia B.A., Shabanowitz J., Hunt D.F., Allis C.D. (2008). Cathepsin L Proteolytically Processes Histone H3 During Mouse Embryonic Stem Cell Differentiation. Cell.

[33] Workman J.L., Kingston R.E. (1998). Alteration of nucleosome structure as a mechanism of transcriptional regulation. Annu. Rev. Biochem..

[34] Pasini D., Hansen K.H., Christensen J., Agger K., Cloos P.A.C., Helin K. (2008). Coordinated regulation of transcriptional repression by the RBP2 H3K4 demethylase and Polycomb-Repressive Complex 2. Genes Dev..

[35] Luger K., Dechassa M.L., Tremethick D.J. (2012). New insights into nucleosome and chromatin structure: An ordered state or a disordered affair?. Nat. Rev. Mol. Cell Biol..

[36] Marmorstein R., Zhou M.M. (2014). Writers and readers of histone acetylation: Structure, mechanism, and inhibition. Cold Spring Harb. Perspect. Biol..

[37] Feingold E.A., Good P.J., Guyer M.S., Kamholz S., Liefer L., Wetterstrand K., Collins F.S., Gingeras T.R., Kampa D., Sekinger E.A. (2004). The ENCODE (ENCyclopedia of DNA Elements) Project. Science.

[38] Mattick J.S., Makunin I.V. (2006). Non-coding RNA. Hum. Mol. Genet..

[39] Choudhuri S. (2010). Small noncoding RNAs: Biogenesis, function, and emerging significance in toxicology. J. Biochem. Mol. Toxicol..

[40] Uchida S., Dimmeler S. (2015). Long noncoding RNAs in cardiovascular diseases. Circ. Res..

[41] Wang K.C., Chang H.Y. (2011). Molecular Mechanisms of Long Noncoding RNAs. Mol. Cell.

[42] Kim T.K., Hemberg M., Gray J.M., Costa A.M., Bear D.M., Wu J., Harmin D.A., Laptewicz M., Barbara-Haley K., Kuersten S. (2010). Widespread transcription at neuronal activity-regulated enhancers. Nature.

[43] Ferguson-Smith A.C., Sasaki H., Cattanach B.M., Surani M.A. (1993). Parental-origin-specific epigenetic modification of the mouse H19 gene. Nature.

[44] Herzing L.B.K., Romer J.T., Horn J.M., Ashworth A. (1997). Xist has properties of the X-chromosome inactivation centre. Nature.

[45] Yap K.L., Li S., Muñoz-Cabello A.M., Raguz S., Zeng L., Mujtaba S., Gil J., Walsh M.J., Zhou M.M. (2010). Molecular Interplay of the Noncoding RNA ANRIL and Methylated Histone H3 Lysine 27 by Polycomb CBX7 in Transcriptional Silencing of INK4a. Mol. Cell.

[46] Astori G., Vignati F., Bardelli S., Tubio M., Gola M., Albertini V., Bambi F., Scali G., Castelli D., Rasini V. (2007). “In vitro” and multicolor phenotypic characterization of cell subpopulations identified in fresh human adipose tissue stromal vascular fraction and in the derived mesenchymal stem cells. J. Transl. Med..

[47] Boyer L.A., Tong I.L., Cole M.F., Johnstone S.E., Levine S.S., Zucker J.P., Guenther M.G., Kumar R.M., Murray H.L., Jenner R.G. (2005). Core transcriptional regulatory circuitry in human embryonic stem cells. Cell.

[48] Okita K., Ichisaka T., Yamanaka S. (2007). Generation of germline-competent induced pluripotent stem cells. Nature.

[49] Rumman M., Dhawan J., Kassem M. (2015). Concise Review: Quiescence in Adult Stem Cells: Biological Significance and Relevance to Tissue Regeneration. Stem Cells.

[50] Saldaña L., Bensiamar F., Vallés G., Mancebo F.J., García-Rey E., Vilaboa N. (2019). Immunoregulatory potential of mesenchymal stem cells following activation by macrophage-derived soluble factors. Stem Cell Res. Ther..

[51] Samsonraj R.M., Raghunath M., Nurcombe V., Hui J.H., van Wijnen A.J., Cool S.M. (2017). Concise Review: Multifaceted Characterization of Human Mesenchymal Stem Cells for Use in Regenerative Medicine. Stem Cells Transl. Med..

[52] Vincent A., Van Seuningen I. (2009). Epigenetics, stem cells and epithelial cell fate. Differentiation.

[53] Piekarz R.L., Bates S.E. (2009). Epigenetic Modifiers: Basic Understanding and Clinical Development. Clin. Cancer Res..

[54] Bibikova M., Chudin E., Wu B., Zhou L., Garcia E.W., Liu Y., Shin S., Plaia T.W., Auerbach J.M., Arking D.E. (2006). Human embryonic stem cells have a unique epigenetic signature. Genome Res..

[55] Atlasi Y., Stunnenberg H.G. (2017). The interplay of epigenetic marks during stem cell differentiation and development. Nat. Rev. Genet..

[56] Mottamal M., Zheng S., Huang T.L., Wang G. (2015). Histone deacetylase inhibitors in clinical studies as templates for new anticancer agents. Molecules.

[57] Buzanska L. (2018). Human Neural Stem Cells: From Generation to Differentiation and Application.

[58] Aguilar-Gallardo C., Simón C. (2013). Cells, stem cells, and cancer stem cells. Semin. Reprod. Med..

[59] Alexanian A.R. (2007). Epigenetic modifiers promote efficient generation of neural-like cells from bone marrow-derived mesenchymal cells grown in neural environment. J. Cell. Biochem..

[60] Chen Q., Shou P., Zheng C., Jiang M., Cao G., Yang Q., Cao J., Xie N., Velletri T., Zhang X. (2016). Fate decision of mesenchymal stem cells: Adipocytes or osteoblasts?. Cell Death Differ..

[61] Hemming S., Cakouros D., Isenmann S., Cooper L., Menicanin D., Zannettino A., Gronthos S. (2014). EZH2 and KDM6A act as an epigenetic switch to regulate mesenchymal stem cell lineage specification. Stem Cells.

[62] Ye L., Fan Z., Yu B., Chang J., Al Hezaimi K., Zhou X., Park N.H., Wang C.Y. (2012). Histone demethylases KDM4B and KDM6B promotes osteogenic differentiation of human MSCs. Cell Stem Cell.

[63] Xu J., Yu B., Hong C., Wang C.Y. (2013). KDM6B epigenetically regulates odontogenic differentiation of dental mesenchymal stem cells. Int. J. Oral Sci..

[64] De Haan G., Gerrits A. (2007). Epigenetic control of hematopoietic stem cell aging: The case of Ezh2. Ann. N. Y. Acad. Sci..

[65] Chou R.H., Yu Y.L., Hung M.C. (2011). The roles of EZH2 in cell lineage commitment. Am. J. Transl. Res..

[66] Caretti G., Di Padova M., Micales B., Lyons G.E., Sartorelli V. (2004). The Polycomb Ezh2 methyltransferase regulates muscle gene expression and skeletal muscle differentiation. Genes Dev..

[67] Sher F., Rößler R., Brouwer N., Balasubramaniyan V., Boddeke E., Copray S. (2008). Differentiation of Neural Stem Cells into Oligodendrocytes: Involvement of the Polycomb Group Protein Ezh2. Stem Cells.

[68] Rosa A., Brivanlou A.H. (2011). A regulatory circuitry comprised of miR-302 and the transcription factors OCT4 and NR2F2 regulates human embryonic stem cell differentiation. EMBO J..

[69] Pursani V., Pethe P., Bashir M., Sampath P., Tanavde V., Bhartiya D. (2017). Genetic and Epigenetic Profiling Reveals EZH2-mediated Down Regulation of OCT-4 Involves NR2F2 during Cardiac Differentiation of Human Embryonic Stem Cells. Sci. Rep..

[70] Villasante A., Piazzolla D., Li H., Gomez-Lopez G., Djabali M., Serrano M. (2011). Epigenetic regulation of Nanog expression by Ezh2 in pluripotent stem cells. Cell Cycle.

[71] Kashyap V., Rezende N.C., Scotland K.B., Shaffer S.M., Persson J.L., Gudas L.J., Mongan N.P. (2009). Regulation of Stem cell pluripotency and differentiation involves a mutual regulatory circuit of the Nanog, OCT4, and SOX2 pluripotency transcription factors with polycomb Repressive Complexes and Stem Cell microRNAs. Stem Cells Dev..

[72] Pan G., Li J., Zhou Y., Zheng H., Pei D. (2006). A negative feedback loop of transcription factors that controls stem cell pluripotency and self-renewal. FASEB J..

[73] Chew J., Tam W., Yeap L., Li P., Ang Y., Lim B., Robson P., Ng H. (2005). Reciprocal transcriptional regulation of Pou5f1 and Sox2 via the Oct4/Sox2 complex in embryonic stem cells. Mol. Cell. Biol..

[74] Bernstein B.E., Mikkelsen T.S., Xie X., Kamal M., Huebert D.J., Cuff J., Fry B., Meissner A., Wernig M., Plath K. (2006). A Bivalent Chromatin Structure Marks Key Developmental Genes in Embryonic Stem Cells. Cell.

[75] Grandy R.A., Whitfield T.W., Wu H., Fitzgerald M.P., VanOudenhove J.J., Zaidi S.K., Montecino M.A., Lian J.B., van Wijnen A.J., Stein J.L. (2016). Genome-Wide Studies Reveal that H3K4me3 Modification in Bivalent Genes Is Dynamically Regulated during the Pluripotent Cell Cycle and Stabilized upon Differentiation. Mol. Cell. Biol..

[76] Challen G.A., Sun D., Jeong M., Luo M., Jelinek J., Berg J.S., Bock C., Vasanthakumar A., Gu H., Xi Y. (2012). Dnmt3a is essential for hematopoietic stem cell differentiation. Nat. Genet..

[77] Huang C., Xu M., Zhu B. (2013). Epigenetic inheritance mediated by histone lysine methylation: Maintaining transcriptional states without the precise restoration of marks?. Philos. Trans. R. Soc. B Biol. Sci..

[78] Reverón-Gómez N., González-Aguilera C., Stewart-Morgan K.R., Petryk N., Flury V., Graziano S., Johansen J.V., Jakobsen J.S., Alabert C., Groth A. (2018). Accurate Recycling of Parental Histones Reproduces the Histone Modification Landscape during DNA Replication. Mol. Cell.

[79] O’Kane C.J., Hyland E.M. (2019). Yeast epigenetics: The inheritance of histone modification states. Biosci. Rep..

[80] Poole R.M. (2014). Belinostat: First global approval. Drugs.

[81] Whittaker S.J., Demierre M.F., Kim E.J., Rook A.H., Lerner A., Duvic M., Scarisbrick J., Reddy S., Robak T., Becker J.C. (2010). Final results from a multicenter, international, pivotal study of romidepsin in refractory cutaneous T-cell lymphoma. J. Clin. Oncol..

[82] Park J.W., Han J.W. (2019). Targeting epigenetics for cancer therapy. Arch. Pharm. Res..

[83] Cavalli G., Heard E. (2019). Advances in epigenetics link genetics to the environment and disease. Nature.

[84] Yoshioka K.I., Matsuno Y., Hyodo M., Fujimori H. (2019). Genomic-destabilization-associated mutagenesis and clonal evolution of cells with mutations in tumor-suppressor genes. Cancers (Basel).

[85] Valkenburg K.C., De Groot A.E., Pienta K.J. (2018). Targeting the tumour stroma to improve cancer therapy. Nat. Rev. Clin. Oncol..

[86] Kelly T.K., De Carvalho D.D., Jones P.A. (2010). Epigenetic modifications as therapeutic targets. Nat. Biotechnol..

[87] Constâncio V., Nunes S.P., Moreira-Barbosa C., Freitas R., Oliveira J., Pousa I., Oliveira J., Soares M., Dias C.G., Dias T. (2019). Early detection of the major male cancer types in blood-based liquid biopsies using a DNA methylation panel. Clin. Epigenet..

[88] Nunes S.P., Moreira-Barbosa C., Salta S., de Sousa S.P., Pousa I., Oliveira J., Soares M., Rego L., Dias T., Rodrigues J. (2018). Cell-free DNA methylation of selected genes allows for early detection of the major cancers in women. Cancers (Basel).

[89] Shi Y.X., Sheng D.Q., Cheng L., Song X.Y. (2019). Current Landscape of Epigenetics in Lung Cancer: Focus on the Mechanism and Application. J. Oncol..

[90] Yao J., Chen J., Li L.-Y., Wu M. (2020). Epigenetic plasticity of enhancers in cancer. Transcription.

[91] Sanaei M., Kavoosi F. (2019). Histone Deacetylases and Histone Deacetylase Inhibitors: Molecular Mechanisms of Action in Various Cancers. Adv. Biomed. Res..

[92] Tortorella S.M., Hung A., Karagiannis T.C. (2015). The Implication of Cancer Progenitor Cells and the Role of Epigenetics in the Development of Novel Therapeutic Strategies for Chronic Myeloid Leukemia. Antioxid. Redox Signal..

[93] Becker M.S., Schmezer P., Breuer R., Haas S.F., Essers M.A., Krammer P.H., Li-Weber M. (2014). The traditional Chinese medical compound Rocaglamide protects nonmalignant primary cells from DNA damage-induced toxicity by inhibition of p53 expression. Cell Death Dis..

[94] Das D., Ghosh S., Maitra A., Biswas N.K., Panda C.K., Roy B., Sarin R., Majumder P.P. (2019). Epigenomic dysregulation-mediated alterations of key biological pathways and tumor immune evasion are hallmarks of gingivo-buccal oral cancer. Clin. Epigenet..

[95] Yu J., Zayas J., Qin B., Wang L. (2019). Targeting DNA methylation for treating triple-negative breast cancer. Pharmacogenomics.

[96] Xu S., Liu H., Wan L., Zhang W., Wang Q., Zhang S., Shang S., Zhang Y., Pang D. (2019). The MS-lincRNA landscape reveals a novel lincRNA BCLIN25 that contributes to tumorigenesis by upregulating ERBB2 expression via epigenetic modification and RNA–RNA interactions in breast cancer. Cell Death Dis..

[97] Nunes S.P., Diniz F., Moreira-Barbosa C., Constâncio V., Silva A.V., Oliveira J., Soares M., Paulino S., Cunha A.L., Rodrigues J. (2019). Subtyping Lung Cancer Using DNA Methylation in Liquid Biopsies. J. Clin. Med..

[98] Moreira-Barbosa C., Barros-Silva D., Costa-Pinheiro P., Torres-Ferreira J., Constâncio V., Freitas R., Oliveira J., Antunes L., Henrique R., Jerónimo C. (2018). Comparing diagnostic and prognostic performance of two-gene promoter methylation panels in tissue biopsies and urines of prostate cancer patients. Clin. Epigenet..

[99] Hu B.B., Wang X.Y., Gu X.Y., Zou C., Gao Z.J., Zhang H., Fan Y. (2019). N6-methyladenosine (m6A) RNA modification in gastrointestinal tract cancers: Roles, mechanisms, and applications. Mol. Cancer.

[100] Chen Y., Zhou C., Sun Y., He X., Xue D. (2019). m ^6^ A RNA modification modulates gene expression and cancer-related pathways in clear cell renal cell carcinoma. Epigenomics.

[101] Orouji E., Peitsch W.K., Orouji A., Houben R., Utikal J. (2020). Oncogenic Role of an Epigenetic Reader of m6A RNA Modification: YTHDF1 in Merkel Cell Carcinoma. Cancers (Basel).

[102] Lin Y., Chen Z., Zheng Y., Liu Y., Gao J., Lin S., Chen S. (2019). MiR-506 Targets UHRF1 to Inhibit Colorectal Cancer Proliferation and Invasion via the KISS1/PI3K/NF-κB Signaling Axis. Front. Cell Dev. Biol..

[103] Shi Y., Wei J., Chen Z., Yuan Y., Li X., Zhang Y., Meng Y., Hu Y., Du H. (2019). Integrative Analysis Reveals Comprehensive Altered Metabolic Genes Linking with Tumor Epigenetics Modification in Pan-Cancer. Biomed. Res. Int..

[104] Dong Z., Pu L., Cui H. (2020). Mitoepigenetics and Its Emerging Roles in Cancer. Front. Cell Dev. Biol..

[105] Martín B., Pappa S., Díez-Villanueva A., Mallona I., Custodio J., Barrero M.J., Peinado M.A., Jordà M. (2020). Tissue and cancer-specific expression of DIEXF is epigenetically mediated by an Alu repeat. Epigenetics.

[106] Yang L., Lei Q., Li L., Yang J., Dong Z., Cui H. (2019). Silencing or inhibition of H3K79 methyltransferase DOT1L induces cell cycle arrest by epigenetically modulating c-Myc expression in colorectal cancer. Clin. Epigenet..

[107] Li S., Chen K., Zhang Y., Barnes S.D., Jaichander P., Zheng Y., Hassan M., Malladi V.S., Skapek S.X., Xu L. (2019). Twist2 amplification in rhabdomyosarcoma represses myogenesis and promotes oncogenesis by redirecting MyoD DNA binding. Genes Dev..

[108] Fachal L., Aschard H., Beesley J., Barnes D.R., Allen J., Kar S., Pooley K.A., Dennis J., Michailidou K., Turman C. (2020). Fine-mapping of 150 breast cancer risk regions identifies 191 likely target genes. Nat. Genet..

[109] Aloia L., McKie M.A., Vernaz G., Cordero-Espinoza L., Aleksieva N., van den Ameele J., Antonica F., Font-Cunill B., Raven A., Aiese Cigliano R. (2019). Epigenetic remodelling licences adult cholangiocytes for organoid formation and liver regeneration. Nat. Cell Biol..

[110] Robertson F.L., Marqués-Torrejón M.A., Morrison G.M., Pollard S.M. (2019). Experimental models and tools to tackle glioblastoma. DMM Dis. Model. Mech..

[111] Abbas M.N., Kausar S., Cui H. (2019). Therapeutic potential of natural products in glioblastoma treatment: Targeting key glioblastoma signaling pathways and epigenetic alterations. Clin. Transl. Oncol..

[112] Schötterl S., Hübner M., Armento A., Veninga V., Wirsik N.M., Bernatz S., Lentzen H., Mittelbronn M., Naumann U. (2017). Viscumins functionally modulate cell motility-associated gene expression. Int. J. Oncol..

[113] Zhou L., Quan Dean J. (2015). Reprogramming the genome to totipotency in mouse embryos. Trends Cell Biol..

[114] Stueve T.R., Marconett C.N., Zhou B., Borok Z., Laird-Offringa I.A. (2016). The importance of detailed epigenomic profiling of different cell types within organs. Epigenomics.

[115] Lilja T., Wallenborg K., Björkman K., Albåge M., Eriksson M., Lagercrantz H., Rohdin M., Hermanson O. (2013). Novel alterations in the epigenetic signature of MeCP2-targeted promoters in lymphocytes of Rett syndrome patients. Epigenetics.

[116] Fernández-Santiago R., Merkel A., Castellano G., Heath S., Raya Á., Tolosa E., Martí M.J., Consiglio A., Ezquerra M. (2019). Whole-genome DNA hyper-methylation in iPSC-derived dopaminergic neurons from Parkinson’s disease patients. Clin. Epigenet..

[117] Zhang F., Kang Y., Wang M., Li Y., Xu T., Yang W., Song H., Wu H., Shu Q., Jin P. (2018). Fragile X mental retardation protein modulates the stability of its m6A-marked messenger RNA targets. Hum. Mol. Genet..

[118] Schnerch A., Rampalii S., Bhatia M. (2013). Histone modification profiling in normal and transformed human embryonic stem cells using micro chromatin immunoprecipitation, scalable to genome-wide microarray analyses. Methods Mol. Biol..

[119] A Phase II Study of Epigenetic Therapy to Overcome Chemotherapy Resistance in Refractory Solid Tumors. https://clinicaltrials.gov/ct2/show/NCT00404508.

[120] Gene Expression Variation and Implant Wound Healing Among Smokers and Diabetics. https://clinicaltrials.gov/ct2/show/NCT01663298?cond=Gene+Expression+Variation+and+Implant+Wound+Healing+Among+Smokers+and+Diabetics&draw=2&rank=1.

[121] Tao H., Li H., Su Y., Feng D., Wang X., Zhang C., Ma H., Hu Q. (2014). Histone methyltransferase G9a and H3K9 dimethylation inhibit the self-renewal of glioma cancer stem cells. Mol. Cell. Biochem..

[122] Hydralazine and Valproate Plus Cisplatin Chemoradiation in Cervical Cancer. https://clinicaltrials.gov/ct2/show/NCT00404326?cond=Hydralazine+and+Valproate+Plus+Cisplatin+Chemoradiation+in+Cervical+Cancer&draw=2&rank=1.

[123] Plummer R., Vidal L., Griffin M., Lesley M., De Bono J., Coulthard S., Sludden J., Siu L.L., Chen E.X., Oza A.M. (2009). Phase I study of MG98, an oligonucleotide antisense inhibitor of human DNA methyltransferase 1, given as a 7-day infusion in patients with advanced solid tumors. Clin. Cancer Res..

[124] Van den Boom V., Maat H., Geugien M., Rodríguez López A., Sotoca A.M., Jaques J., Brouwers-Vos A.Z., Fusetti F., Groen R.W.J., Yuan H. (2016). Non-canonical PRC1.1 Targets Active Genes Independent of H3K27me3 and Is Essential for Leukemogenesis. Cell Rep..

[125] Study of Azacitidine in Adult Taiwanese Subjects With Higher-Risk Myelodysplastic Syndromes (MDS). https://clinicaltrials.gov/ct2/show/NCT01201811?cond=Study+of+Azacitidine+in+Adult+Taiwanese+Subjects+With+Higher-Risk+Myelodysplastic+Syndromes+%28MDS%29&draw=2&rank=1.

[126] Torres C.M., Biran A., Burney M.J., Patel H., Henser-Brownhill T., Cohen A.H.S., Li Y., Ben-Hamo R., Nye E., Spencer-Dene B. (2016). The linker histone H1.0 generates epigenetic and functional intratumor heterogeneity. Science.

[127] Vidaza to Restore Hormone Thx Prostate. https://clinicaltrials.gov/ct2/show/NCT00384839.

[128] A Study of Venetoclax in Combination With Azacitidine Versus Azacitidine in Treatment Naïve Subjects With Acute Myeloid Leukemia Who Are Ineligible for Standard Induction Therapy. https://clinicaltrials.gov/ct2/show/NCT02993523.

[129] A Trial of Epigenetic Priming in Patients With Newly Diagnosed Acute Myeloid Leukemia. https://clinicaltrials.gov/ct2/show/NCT03164057.

[130] Azacytidine Prior to in Vivo T-cell Depleted Allo Stem Cell Transplant for Patients With Myeloid Malignancies in CR. https://clinicaltrials.gov/ct2/show/NCT02497404?cond=Azacytidine+Prior+to+in+Vivo+T-cell+Depleted+Allo+Stem+Cell+Transplant+for+Patients+With+Myeloid+Malignancies+in+CR&draw=2&rank=1.

[131] Diagnosis of RSTS: Identification of the Acetylation Profiles as Epigenetic Markers for Assessing Causality of CREBBP Variants. https://clinicaltrials.gov/ct2/show/NCT04122742?cond=Diagnosis+of+RSTS%3A+Identification+of+the+Acetylation+Profiles+as+Epigenetic+Markers+for+Assessing+Causality+of+CREBBP+Variants&draw=2&rank=1.

[132] DNA Methylation in Allogeneic Hematopoietic Stem Cell Transplantation. https://clinicaltrials.gov/ct2/show/NCT03871296?cond=DNA+Methylation+in+Allogeneic+Hematopoietic+Stem+Cell+Transplantation&draw=2&rank=1.

[133] EPIgenetics and in Vivo Resistance of Chronic Myeloid Leukemia Stem Cells to Tyrosine Kinase Inhibitors. https://clinicaltrials.gov/ct2/show/NCT03481868?cond=EPIgenetics+and+in+Vivo+Resistance+of+Chronic+Myeloid+Leukemia+Stem+Cells+to+Tyrosine+Kinase+Inhibitors&draw=2&rank=1.

[134] Genetic and Epigenetic Basis of Chronic Wounds. https://clinicaltrials.gov/ct2/show/NCT03793062?cond=Genetic+and+Epigenetic+Basis+of+Chronic+Wounds&draw=2&rank=1.

[135] Phase II Anti-PD1 Epigenetic Therapy Study in NSCLC. https://clinicaltrials.gov/ct2/show/NCT01928576.

[136] The Efficacy and Safety of Oral Azacitidine Plus Best Supportive Care Versus Placebo and Best Supportive Care in Subjects With Red Blood Cell (RBC) Transfusion-Dependent Anemia and Thrombocytopenia Due to International Prognostic Scoring System (IPSS). https://clinicaltrials.gov/ct2/show/NCT01566695.

[137] Wong E., Juneja S. (2015). Acute myeloid leukaemia and myelodysplastic syndromes with 50% or greater erythroblasts: A diagnostic conundrum. Pathology.

[138] Diesch J., Zwick A., Garz A.K., Palau A., Buschbeck M., Götze K.S. (2016). A clinical-molecular update on azanucleoside-based therapy for the treatment of hematologic cancers. Clin. Epigenet..

[139] Schuh A.C., Döhner H., Pleyer L., Seymour J.F., Fenaux P., Dombret H. (2017). Azacitidine in adult patients with acute myeloid leukemia. Crit. Rev. Oncol. Hematol..

[140] Baylin S.B. (2005). DNA methylation and gene silencing in cancer. Nat. Clin. Pract. Oncol..

[141] Sundar R., Cho B.C., Brahmer J.R., Soo R.A. (2015). Nivolumab in NSCLC: Latest evidence and clinical potential. Ther. Adv. Med. Oncol..

[142] Arce C., Segura-Pacheco B., Perez-Cardenas E., Taja-Chayeb L., Candelaria M., Dueñnas-Gonzalez A. (2006). Hydralazine target: From blood vessels to the epigenome. J. Transl. Med..

[143] Cervera E., Candelaria M., López-Navarro O., Labardini J., Gonzalez-Fierro A., Taja-Chayeb L., Cortes J., Gordillo-Bastidas D., Dueñas-González A. (2012). Epigenetic therapy with hydralazine and magnesium valproate reverses imatinib resistance in patients with chronic myeloid leukemia. Clin. Lymphoma Myeloma Leuk.

[144] Candelaria M., Gallardo-Rincón D., Arce C., Cetina L., Aguilar-Ponce J.L., Arrieta O., González-Fierro A., Chávez-Blanco A., de la Cruz-Hernández E., Camargo M.F. (2007). A phase II study of epigenetic therapy with hydralazine and magnesium valproate to overcome chemotherapy resistance in refractory solid tumors. Ann. Oncol. Off. J. Eur. Soc. Med. Oncol..

[145] De La Cruz-Hernández E., Pérez-Cárdenas E., Contreras-Paredes A., Cantú D., Mohar A., Lizano M., Dueñas-González A. (2007). The effects of DNA methylation and histone deacetylase inhibitors on human papillomavirus early gene expression in cervical cancer, an in vitro and clinical study. Virol. J..

[146] Hogarth L., Hall A.G., Skitt L., Coulthard S.A. (2005). Epigenetic effects of the thiopurine drugs. Cancer Res..

[147] Liew E., Owen C. (2011). Familial myelodysplastic syndromes: A review of the literature. Haematologica.

[148] Garcia-Manero G., Almeida A., Giagounidis A., Platzbecker U., Garcia R., Voso M.T., Larsen S.R., Valcarcel D., Silverman L.R., Skikne B. (2016). Design and rationale of the QUAZAR Lower-Risk MDS (AZA-MDS-003) trial: A randomized phase 3 study of CC-486 (oral azacitidine) plus best supportive care vs placebo plus best supportive care in patients with IPSS lower-risk myelodysplastic syndromes and po. BMC Hematol..

[149] Chou W.C., Yeh S.P., Hsiao L.T., Lin S.F., Chen Y.C., Chen T.Y., Laille E., Galettis A., Dong Q., Songer S. (2017). Efficacy, safety, and pharmacokinetics of subcutaneous azacitidine in Taiwanese patients with higher-risk myelodysplastic syndromes. Asia Pac. J. Clin. Oncol..

[150] Calvanese V., Lara E., Kahn A., Fraga M.F. (2009). The role of epigenetics in aging and age-related diseases. Ageing Res. Rev..

[151] Bacalini M.G., Gentilini D., Boattini A., Giampieri E., Pirazzini C., Giuliani C., Fontanesi E., Scurti M., Remondini D., Capri M. (2015). Identification of a DNA methylation signature in blood cells from persons with down syndrome. Aging (Albany N.Y.).

